# Public Health, Sanitation and Vital Statistics

**Published:** 1896-05

**Authors:** Franklin C. Gram

**Affiliations:** Buffalo, N. Y., Registrar of vital statistics, Department of Health


### PUBLIC HEALTH, SANITATION AND VITAL STATISTICS.

By FRANKLIN C. GRAM, M. D., Buffalo, N. Y.,
Registrar of vital statistics, Department of Health.

IT MAY not be uninteresting to physicians to know that despite the ever-increasing population of Buffalo the death-rate continues steadily to decrease. Not only is this the case with the rate, but also in the actual number of deaths. This fact has undoubtedly manifested itself to most practitioners when they made a comparison between their present and past book accounts, and no small number have made investigations as to the cause. It is often as important to know why a patient recovered as it is to know why another one died ; and so, in this case, it may be of as much interest to know the reason for the exceptional healthfulness enjoyed by our city as it would be to determine the cause and source of an epidemic.

Sanitarians in various parts of this country have been attracted by the published official reports and Health Commissioner Wende has received inquiries asking him to explain this "unprecedented death-rate," as one of the correspondents tersely put it.

Briefly stated, a synopsis for the past five years shows the following :

| Year. | Deaths. | Rate. |
| --- | --- | --- |
| 1891 | 6,001 | 23.48 |
| 1892 | 5,697 | 19.98 |
| 1893 | 5,711 | 19.03 |
| 1894 | 5,280 | 16.76 |
| 1895 | 4,684 | 13.95 |

In 1891 the city's population was 255,664 and according to the police census it was 335,709 in 1895.

Undoubtedly the cause of this great reduction in the number of deaths is mainly due to the decrease in communicable diseases, including consumption, the percentage of deaths in these having decreased from 7.42 in 1891 to 4.04 in 1895.

The Health Department is frequently criticised as being unnecessarily severe in its vigilance over contagious diseases, in looking after the sanitary conditions of dwellings, or in preventing the intermingling of unhealthy or exposed children or adults with healthy persons in schools, places of business or elsewhere. Extraordinary as it may seem, not a few physicians have been among this class of critics, and one of their arguments advanced is that in former years there were fewer contagious diseases although the public had comparatively little or no protection from them. Such arguments do not hold good, however, as the public records show an entirely opposite state of affairs.

Consumption is and will remain the principal cause of death so long as the public declines to be educated to the proper methods which may be employed for its prevention. Gradually physicians are beginning to be more prompt in reporting their cases of tuberculosis, and more are constantly availing themselves of the opportunity to have examinations of sputa made without charge by the Health Department, but it is remarkable to see the number who request that the patient be kept in ignorance of the true nature of the disease and that the circular of instruction be given to the physician for delivery to the family.

Thus far the present year looks very encouraging, as will be seen by the following comparison with last year of the number of deaths and a few of the principal diseases :

| Year. | Month. | Deaths. | Rate. | Under five years. | Consumption, reported. | Consumption, deaths. | Diphtheria, reported. | Diphtheria, deaths. | Diph. Croup, reported. | Diph. Croup, deaths. | Membr. Croup, reported | Membr. Croup, deaths | Scarlet Fever, reported. | Scarlet Fever, deaths. | Typhoid Fever, reported. | Typhoid Fever, deaths. |
|---|---|---|---|---|---|---|---|---|---|---|---|---|---|---|---|---|
| 1896 | January ...... | 327 | 11.68 | 98 | 28 | 38 | — | 16 | 6 | 6 | 18 | 16 | 26 | — | 19 | 5 |
| 1895 | January ...... | 372 | 14.17 | 149 | — | 44 | 80 | 25 | 4 | 4 | 4 | 3 | 78 | 2 | 24 | 5 |
| 1896 | February..... | 297 | 10 61 | 84 | 38 | 43 | — | 7 | 3 | 3 | 10 | 8 | 21 | 2 | 16 | 3 |
| 1895 | February. .. | 409 | 15.58 | 149 | — | 40 | 55 | 13 | — | — | 2 | 2 | 58 | 3 | 17 | 3 |
| 1896 | March ....... | 324 | 11.58 | 96 | 45 | 40 | — | 12 | 2 | 2 | 7 | 7 | 41 | 2 | 20 | 4 |
| 1895 | March ....... | 416 | 15.84 | 151 | — | 36 | 59 | 8 | 2 | 2 | 8 | 7 | 37 | 2 | 4 | 6 |

